# Can Pre-Operative Neutrophil-to-Lymphocyte Ratio (NLR) Help Predict Non-Metastatic Renal Carcinoma Recurrence after Nephrectomy? (UroCCR-61 Study)

**DOI:** 10.3390/cancers14225692

**Published:** 2022-11-19

**Authors:** Clément Allenet, Clément Klein, Benjamin Rouget, Gaëlle Margue, Grégoire Capon, Eric Alezra, Peggy Blanc, Vincent Estrade, Franck Bladou, Grégoire Robert, Jean-Christophe Bernhard

**Affiliations:** 1 Department of Urology, Bordeaux Pellegrin University Hospital,33000 Bordeaux, France; 2 Department of Health Sciences, University of Bordeaux, 33000 Bordeaux, France; 3 Department of Urology, Robert Boulin Hospital, 33500 Libourne, France

**Keywords:** renal cell carcinoma, localized RCC, neutrophil–lymphocyte ratio, prognostic factor, bio-marker

## Abstract

**Simple Summary:**

Accurately predicting the recurrence of non-metastatic renal cell carcinoma following surgery is of utmost importance to guide follow-up recommendations, as well as adjuvant treatment indications. In this study, we looked at the prognostic role of the pre-operative neutrophil-to-lymphocyte ratio (NLR) in this setting. We found that the pre-operative NLR was an interesting bio-marker to predict disease recurrence and death. It is all the more interesting because it is inexpensive and easy to implement. Furthermore, it could increase the performance of the UISS classification (which is actually recommended to predict the risk of recurrence after nephrectomy) and allow to individualize a group of patients at very low risk who could benefit from a lightened follow-up.

**Abstract:**

Recent studies suggested that the neutrophil-to-lymphocyte ratio (NLR) could play a key role in tumor initiation, progression and response to treatments. The main objective was to assess the prognostic value of the pre-operative NLR on recurrence-free survival (RFS) in patients with non-hereditary localized renal cell carcinoma. From the UroCCR database (NCT03293563), factors influencing the disease recurrence of consecutive patients who underwent nephrectomy for cT1-T4 N0M0 were analyzed using multi-variate cox regression and log-rank methods. We included 786 patients, among which 135 (17.2%) experienced a recurrence at a median time of 23.7 [8.5–48.6] months. RFS for patients with a pre-operative NLR of <2.7 was 94% and 88% at 3 and 5 years, respectively, versus 76% and 63% for patients with a NLR of ≥2.7 (*p* < 0.001, log-rank test). To predict the risk of post-operative recurrence, the NLR was combined with the UCLA integrated staging system (UISS), and we defined four groups of the UroCCR-61 predictive model. The RFS rates at 3 and 5 years were 100% and 97% in the very-low-risk group, 93% and 86% in the low-risk group, 78% and 68% in the intermediate-risk group and 63% and 46% in the high-risk group (*p* < 0.0001). The pre-operative NLR seems to be an inexpensive and easily accessible prognostic bio-marker for non-metastatic RCCs.

## 1. Introduction

Currently, the search for predictive bio-markers of response to various anti-tumor treatments is a major concern in many cancers, including renal cell carcinoma (RCC). Although a large proportion of localized RCCs have a favorable prognosis, it is important to emphasize that the risk of relapse after nephrectomy for localized RCC concerns between 30% and 40% of patients within a median of 15 months post-operatively [1]. It, therefore, seems essential to better target this population of patients at risk of recurrence in order to optimize their follow-up with the choice of a possible additional post-operative treatment [2].

For more than twenty years, the UCLA integrated staging system (UISS) has been used to predict the risk of recurrence after nephrectomy. This model classifies localized tumors as being at low, intermediate and high risk of recurrence with a 5-year recurrence-free survival (RFS) of 92%, 67% and 44%, respectively [3]. However, this score, based on histological and clinical data, can be misleading due to the sometimes-unpredictable course of RCC linked to a large intra-tumor biological heterogeneity. Therefore, new prognostic factors need to be identified.

The systemic inflammatory response, including the neutrophil-to-lymphocyte ratio (NLR), which is often used as an indicator, has been shown to play a key role in tumor initiation and progression, and may influence the response to treatments (e.g., chemotherapy and immunotherapy) [4,5]. An elevated pre-operative NLR has been reported to be predictive of poor outcomes in a wide range of cancers, including colorectal, breast, lung and RCC [6]. Recently, Nunno et al. found that an elevated NLR was negatively associated with overall survival (OS) and progression-free survival (PFS) in RCCs [7]. The advantage of this bio-marker is that it is inexpensive and easily accessible, as it is routinely performed.

Nonetheless, data on the prognostic role of the pre-operative NLR for localized and locally advanced RCCs are mostly from retrospective studies with a limited number of patients [8,9,10].

Finally, to date, no study has compared the NLR to the UISS currently recommended by EAU guidelines to predict the risk of post-operative recurrence.

The aim of this study was to evaluate, in a large contemporary cohort, the interest in the NLR as a prognostic bio-marker in localized and locally advanced sporadic RCC (T1-4N0M0).

## 2. Materials and Methods

### 2.1. Study Design

All patients were prospectively enrolled in the UroCCR multi-centric database (CNIL DR 2013-206; NCT03293563). We conducted a mono-centric retrospective analysis of all consecutive patients who underwent radical or partial nephrectomy for a newly diagnosed cT1-T4 N0M0 non-hereditary RCC according to the 2017 TNM classification [11] between May 2000 and January 2019. Patients who received a neo-adjuvant or adjuvant treatment and patients with synchronous metastatic disease (N+ or M+) at the time of diagnosis were not included. The patients were classified according to the UISS. For the early detection of possible recurrence, all patients were followed-up with using computerized tomography according to current recommendations [12].

### 2.2. Study Objectives and Endpoints

The main objective was to assess the prognostic value of the pre-operative NLR on RFS in patients with localized RCC. The primary endpoint was disease recurrence, defined as the detection of a new enhancing lesion in the surgical bed of the original nephrectomy site, contralateral kidney or regional lymph node, or the detection of a lesion in a distant site or in a non-regional retro-peritoneal lymph node.

Secondary objectives were to evaluate the prognostic value of the pre-operative NLR on OS, and to assess the prognostic value of the pre-operative NLR combined with the UISS score on RFS, describing, thence, the UroCCR-61 predictive model. Finally, we described the NLR at the time of recurrence. Overall survival referred to the time between surgery and death, regardless of the cause.

### 2.3. Statistical Analysis

Student’s t-test and chi-square test were used for continuous and categorical variables, respectively. The significance level was set at 0.05 for all statistical tests and *p*-values were two sided.

An area under receiver operator characteristic (AUROC) curve was used to determine the NLR cut-off point with the best sensitivity and specificity for the prediction of recurrence. The cut-off point of 2.7 was then checked using the log-rank test.

A uni-variate and multi-variate Cox proportional hazards regression analysis addressed time to cancer recurrence. The hazard ratio (HR), estimated with the Cox analysis, was reported as a relative risk, with a corresponding 95% confidence interval (CI). The Kaplan–Meier method was utilized to assess the survival curves (RFS and OS), and the statistical significance was determined with the log-rank test.

In order to evaluate whether the association of the pre-operative NLR with the UISS could improve the prognostic performance of the UISS alone, we stratified the UISS risk groups according to the NLR. Four UroCCR-61 groups were obtained: very-low-risk group = UISS low-risk and NLR < 2.7; low-risk group = UISS intermediate-risk and NLR < 2.7; intermediate-risk group = UISS low- and intermediate-risk and NLR ≥ 2.7 together with UISS high-risk and NLR < 2.7; and high-risk group = UISS high-risk and NLR ≥ 2.7. To evaluate the prognostic performance of our model, AUROCs were calculated.

Data analysis was performed using R Foundation for Statistical Computing, Vienna, Austria (version 4.0.0) software.

## 3. Results

### 3.1. Baseline Characteristics of the Study Population According to Recurrence or Not

According to our study criteria, 786 patients were included, among which 135 (17.2%) experienced a recurrence at a median time of 23.7 [8.5–48.6] months.

The mean age was 60.9 ± 13.5; 548 (69.7%) patients were male and 238 (30.3%) were female (Table 1). Patients in the recurrence group were older (64 vs. 60.2; *p* = 0.002) and had a significantly higher ECOG-PS score (*p* = 0.01).

The mean pre-operative tumor size was higher in the recurrence group (68.1 vs. 44.2; *p* < 0.001). Tumor complexity assessed with the RENAL and PADUA scores were higher in the recurrence group with, respectively, 8.9 vs. 7.8 and 10 vs. 9.2 (*p* < 0.001).

The mean pre-operative NLR was 2.8 ± 2.4, and was significantly higher in the recurrence group (4.2 vs. 2.5; *p* < 0.001).

Regarding pathological characteristics, 496 (63.1%), 52 (6.6%), 231 (29.4%) and 7 (0.9%) patients had pT1,2,3 and 4 stages, respectively, and 32 (4.1%) had positive surgical margins. The proportion of pT stages of ≥3 was higher in the recurrence group (56.3% vs. 24.9%). Three hundred and ninety-five (51%) patients had high-grade tumors (3 + 4) according to Fuhrman. The high-grade rate was significantly higher in the recurrence group (75.7% vs. 45.7%; *p* < 0.001).

Respectively, 249 (32.2%), 430 (55.5%) and 95 (12.3%) patients were stratified into low, moderate and high risk according to the UISS classification. The proportion of UISS high-risk patients was 28.9% in the recurrence group, compared to 8.8% in the non-recurrence group (*p* < 0.001).

### 3.2. Prognostic Value of NLR on Recurrence-Free Survival

The median follow-up from surgery was 48 [28.2–70.9] months. Among patients who experienced a recurrence, 35 (25.9%) and 18 (13.3%) had ipsi-lateral and contra-lateral kidney recurrences, respectively. One hundred and eight (80%) patients experienced a disease progression (Table 2).

In the uni-variate analysis, an age of ≥61, a pre-operative NLR of ≥2.7, radical nephrectomy, open approach, pT stage of ≥3, a high Fuhrman grade (3–4), clear cell RCC, sarcomatoid features, microvascular invasion and positive surgical margins were associated with recurrence.

In the multi-variate analysis, only a pre-operative NLR of ≥2.7 (2.89 [1.94–4.31]; *p* < 0.001), open approach, pT stage of ≥3, clear cell RCC and sarcomatoid features remained significant (Table 3).

**Figure 1:**
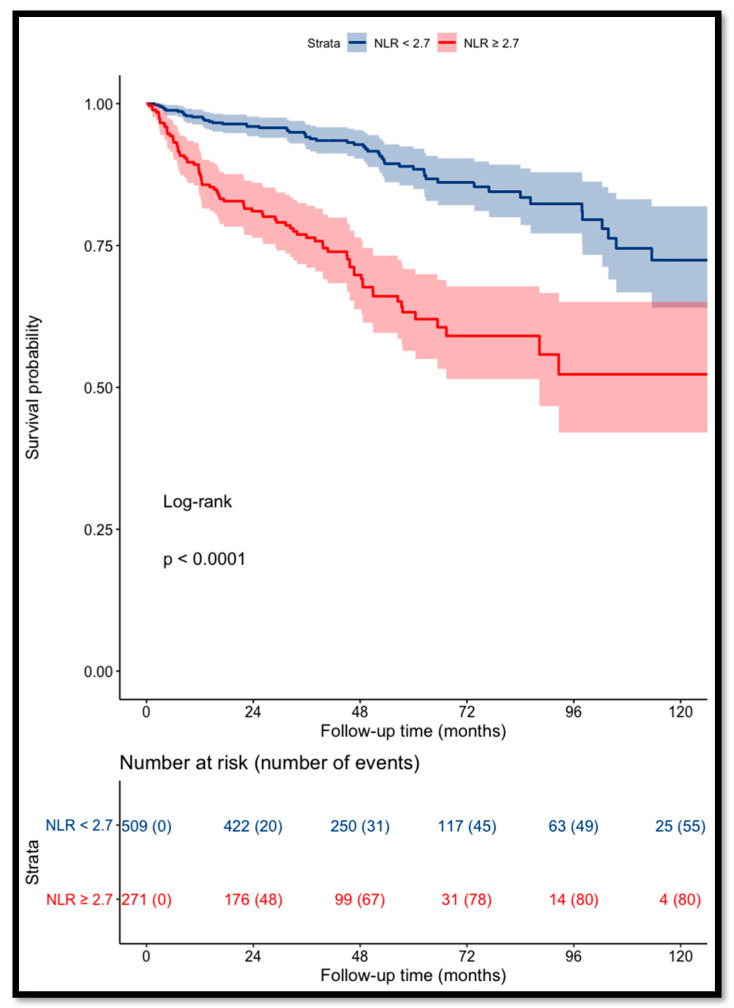
Kaplan–Meier curves for RCC patients’ recurrence-free survival categorized using NLR (cut-off value = 2.7).

The recurrence-free survival for patients with a pre-operative NLR of <2.7 was 94% and 88% at 3 and 5 years, respectively, versus 76% and 63% for patients with a NLR of ≥2.7 (*p* < 0.001, log-rank test) (Figure 1).

### 3.3. Prognostic Value of NLR on Overall Survival

During follow-up, 91 (11.6%) patients died, with 54 (6.9%) cancer-specific deaths and 37 (4.7%) non-cancer-related deaths.

**Figure 2:**
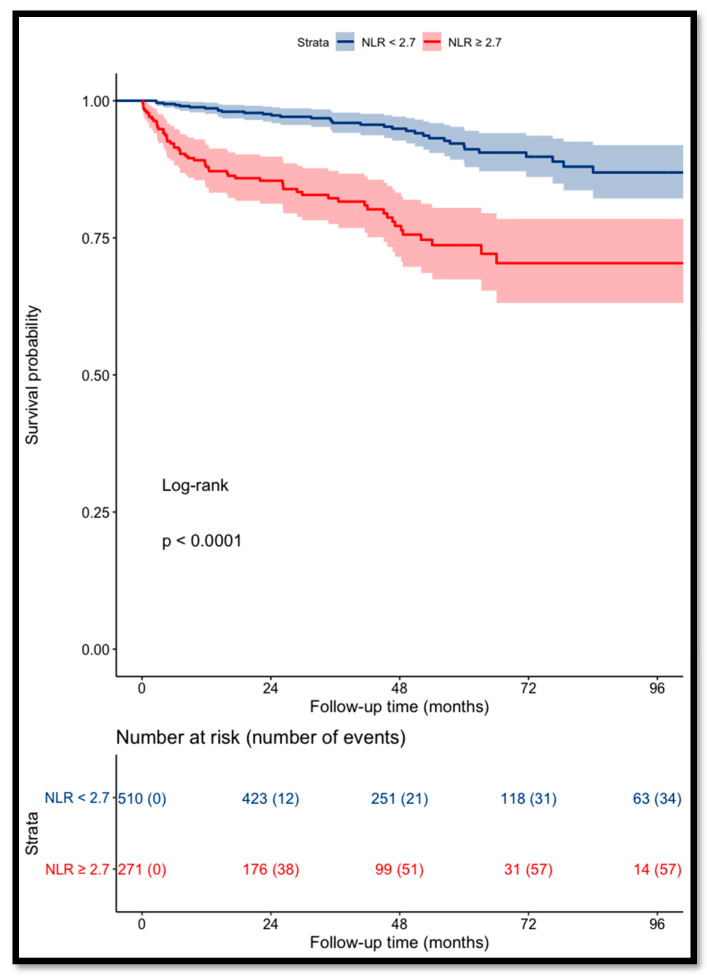
Kaplan–Meier curves for RCC patients’ overall survival categorized using NLR (cut-off value = 2.7).

Patients with a NLR of ≥2.7 had a significant increase in the risk of death (*p* < 0.001) (Figure 2).

The OS for patients with a pre-operative NLR of <2.7 was 97% and 93% at 3 and 5 years, respectively, versus 85% and 77% for patients with a NLR of ≥2.7.

### 3.4. Prognostic Value of Pre-operative NLR Combined with UISS Score on Recurrence-Free Survival: UroCCR-61 Predictive Model

**Figure 3:**
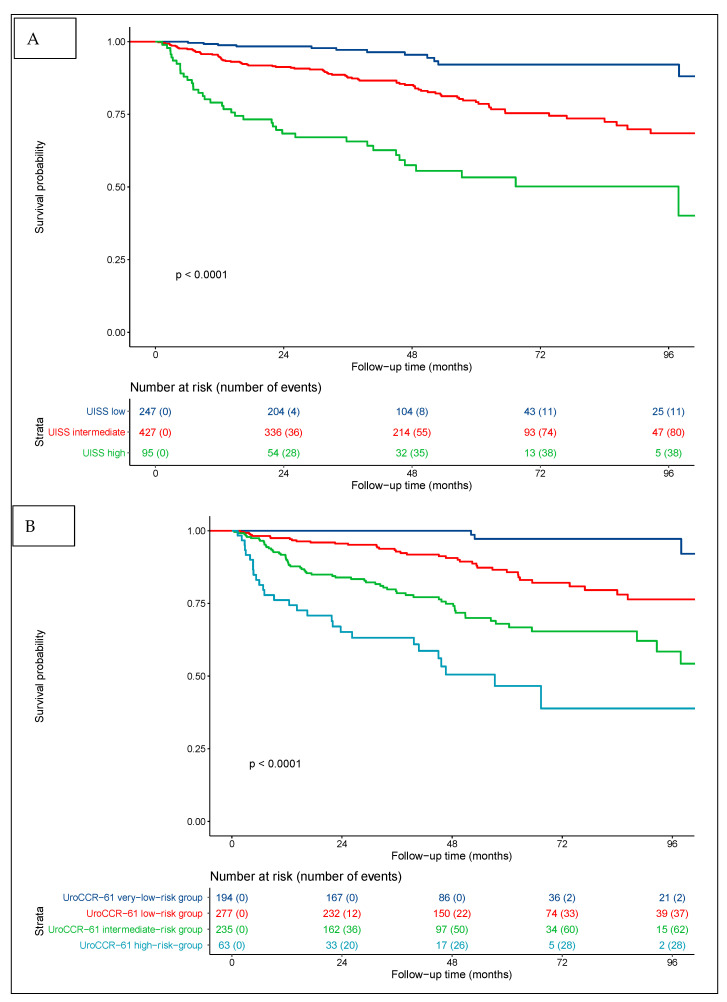
Kaplan–Meier curves for RCC patients’ RFS categorized using UISS score (**A**) and UISS score + pre-operative NLR = UroCCR-61 predictive model (**B**). UroCCR-61 very-low-risk group = UISS low-risk and NLR < 2.7; UroCCR-61 low-risk group = UISS intermediate-risk and NLR < 2.7; UroCCR-61 intermediate-risk group = UISS low- and intermediate-risk and NLR ≥ 2.7 together with UISS high-risk and NLR < 2.7; UroCCR-61 high-risk group = UISS high-risk and NLR ≥ 2.7.

Regarding the UISS score alone, the RFS rates at 3 and 5 years were 97% and 91% for low risk, 87% and 78% for intermediate risk, and 65% and 53% for high risk (*p* < 0.0001) (Figure 3A). The combination of the pre-operative NLR and UISS defined the four groups of the UroCCR-61 predictive model, whose RFS curves are shown in Figure 3B. The RFS rates at 3 and 5 years were 100% and 97% in the very-low-risk group, 93% and 86% in the low-risk group, 78% and 68% in the intermediate-risk group and 63% and 46% in the high-risk group (*p* < 0.0001).

**Figure 4:**
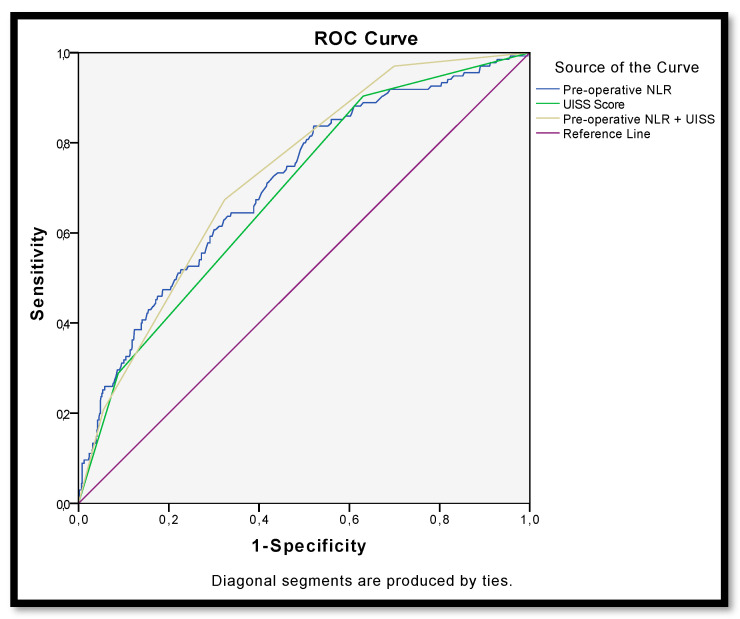
ROC curve for recurrence after surgery for non-metastatic renal carcinoma according to pre-operative NLR (AUC = 0.713), UISS (AUC = 0.692) and NLR + UISS = UroCCR-61 predictive model (AUC = 0.733).

Furthermore, the addition of the NLR to the UISS classification overcame the performance of the UISS in predicting disease recurrence (AUC = 0.733 vs. 0.692) (Figure 4).

### 3.5. NLR at the Time of Recurrence

Among patients with recurrence, the mean NLR at the time of recurrence was 4.9 ± 3.7. It was significantly higher than in patients who did not experienced recurrence with a NLR of 2.9 ± 4.9 at last follow-up (*p* < 0.001).

## 4. Discussion

Many studies have demonstrated the role of local and systemic inflammation in the development and progression of cancer [13]. The study of inflammation bio-markers, such as neutrophils, lymphocytes, CRP and platelets, has shown some correlation with poor oncological prognosis in several cancers (gynecological, digestive and thoracic).

In this study, we evaluated the interest in the NLR as a prognostic bio-marker in localized RCC (T1-4N0M0). Numerous studies focused on the NLR as a prognostic bio-marker, and an elevated NLR was described as a predictive factor of poor outcome in a wide range of cancers [6]. Although it is difficult to determine whether ipsi-lateral and contra-lateral relapses have the same biological determinants as other site metastases, our main endpoint was disease recurrence that combined local relapse and distant progression. Actually, in an attempt to better guide post-operative localized RCC follow-up, we considered it relevant to predict any new oncological event to be diagnosed.

In our study, the NLR cut-off was set at 2.7, as previously described in localized RCC [14,15]. Indeed, it was between 1.7 and 5 [9,10,16], although, in the majority of studies, it was set at 3 ± 0.3. All studies showed a significant prognostic role of the NLR on RFS, except for two studies, for which the NLR threshold was over four [10]. In the case of a pre-operative NLR of ≥2.7, 3 and 5 years RFS and OS were, respectively, reported at 76%, 63%, 85% and 77%. In 2010, Ohno et al. reported on their retrospective study on 250 patients with non-metastatic RCCs, with 5- and 10-year RFS rates for patients with a pre-operative NLR of ≥2.7 of 77.9% and 58.4%, compared to 93.7% and 79.8% for those with a NLR of <2.7 (*p* < 0.001) [17].

Similarly, in their retrospective study on 327 patients, Wen et al. found that a pre-operative NLR of >1.7 was associated with poor RFS (HR = 1.714; 95% IC [1.092–2.691]; *p* = 0.019) and OS (HR = 1.674; 95% CI [1.103–2.539]; *p* = 0.015) [18]. For De Martino et al., a pre-operative NLR of >2.7 was a prognostic factor for recurrence, but not for OS [19]. Boissier et al. published a systematic review in 2017 including six studies reporting on the prognostic value of the NLR in localized RCC. A high pre-operative NLR was significantly associated with an increased risk of recurrence (HR = 1.63 [1.15–2.29]), but no significant association was found for OS [20]. Recently, Di Nunno et al. published a systematic review and meta-analysis about the prognostic role of the NLR in RCCs. Twenty-one studies with 5531 patients were included. They found that, in non-metastatic patients, a higher NLR resulted in worse OS and PFS, with a pooled HR of 1.57 (95% CI [1.27–1.94]; *p* < 0.0001) and 1.52 (95% CI [1.23–1.87]; *p* < 0.0001) [7].

For the first time in the literature, the value of combining a standard prognostic classification, such as the UISS with the NLR for RFS prediction, was assessed. The addition of the NLR to the UISS classification improved the prediction of recurrence. According to our results, UISS low-risk patients with a pre-operative NLR of ≥2.7 had a worse RFS than intermediate-risk patients with a NLR of <2.7. This also applied to intermediate-risk patients with a NLR of ≥2.7, who had a worse RFS than the high-risk patients.

Most importantly, the combination of the NLR with the UISS classification allowed for the identification of a “very-low”-risk group (UroCCR-61 very-low-risk group) among the UISS low-risk group. According to the UISS classification, the 3- and 5-year RFS in the low-risk group with a pre-operative NLR of <2.7 was 100% and 97%. The very-low-risk group represented 209 patients, i.e., 26.5% of the entire cohort. Thus, we truly believe that when using our UroCCR-61 predictive model, follow-up guidance should be significantly lightened for more than a quarter of non-metastatic RCC patients. The EAU guidelines [21] recommend that patients in the UISS low-risk group should undergo five CT scans during their follow-up (one per year). According to our findings, in the very-low-risk group, no oncological control seemed to be needed before post-operative year 5. Such a recommendation of a reduction in post-operative monitoring would have a significant psychological impact for these patients. It could also limit the radiation exposure due to CT scans and play an economical role, reducing costs of follow-ups.

In contrast, patients in the UISS high-risk group with a pre-operative NLR of ≥2.7 (UroCCR-61 predictive model group four) had a very poor 5-year RFS of 46%. These patients could be considered at “very high” risk of disease recurrence, and could potentially represent excellent candidates for adjuvant immune checkpoint inhibitors. Indeed, from a recent report on post-nephrectomy adjuvant pembrolizumab by Choueiri et al. [2], it looked as if the poorer the prognosis, the higher the benefit. One could argue that failure in the accurate selection of truly high-risk patients may play a role in the negative results of the CheckMate-914, InMotion 010 and PROSPER trials, as recently reported.

Our results could be helpful for easily enhancing the post-operative prognostic estimation, follow-up guidance and patient information. Moreover, the NLR at the time of recurrence suggested that this parameter could also be a diagnostic bio-marker of recurrence. A larger multi-centric external validation study with a long-term follow-up would be needed to definitely update urological guidelines.

Apart from the biases linked to the retrospective analysis, this study’s main limitations were the mono-centric design and the mid-term follow-up. On the other hand, major strengths of this study were the representativeness of the results in the contemporary general population, the large number of patients prospectively included and followed in a national database (786 patients, which represented the second most important cohort published) and a, consequently, very low number of missing data. Moreover, our results are applicable to all histological sub-types, even if a sub-group analysis with a histological subtype could be interesting to perform on a larger cohort.

## 5. Conclusions

According to our results, the pre-operative NLR seems to be an inexpensive and easily accessible prognostic bio-marker for localized RCCs. In combination with the UISS classification, it could increase the predictive performance of the UISS to predict recurrence, and the use of the UroCCR-61 predictive model could allow for a reduction in post-operative monitoring for more than 25% of patients who underwent surgery for non-metastatic RCC. A larger multi-centric external validation study with a long-term follow-up is necessary to implement the pre-operative NLR as a predictive bio-marker after nephrectomy for localized RCCs in international guidelines.

## Figures and Tables

**Table 1:** Demographic, biologic and pathological characteristics of the cohort.

	*n*=	Overall Cohort (*n* = 786)	Recurrence (*n* = 135)	No Recurrence (*n* = 651)	*p*-Value
**Age, years** ± SD	786	60.9 ± 13.5	64 ± 12.4	60.2 ± 13.6	**0.002**
**Gender, *n* (%)**	786				0.9
Male		548 (69.7%)	94 (69.6%)	454 (69.7%)	
Female		238 (30.3%)	41 (30.4%)	197 (30.3%)	
**ECOG, *n* (%)**	783				**0.01**
0		558 (71.3%)	83 (61.5%)	475 (73.3%)	
1		161 (20.6%)	41 (30.4%)	120 (18.5%)	
2		58 (7.4%)	9 (6.6%)	49 (7.6%)	
3		6 (0.7%)	2 (1.5%)	4 (0.6%)	
**Symptoms, *n* (%)**	782				**<0.001**
Incidental		556 (71.1%)	77 (57.9%)	479 (73.8%)	
Local		162 (20.7%)	35 (26.3%)	127 (19.6%)	
General		64 (8.2%)	21 (15.8%)	43 (6.6%)	
**Pre-operative NLR** ± SD	786	2.8 ± 2.4	4.2 ± 4	2.4 ± 1.7	**<0.001**
**Type of treatment, *n* (%)**	786				**<0.001**
Partial nephrectomy		517 (65.8%)	55 (40.7%)	462 (71%)	
Radical nephrectomy		269 (34.2%)	80 (59.3%)	189 (29%)	
**Surgical approach, *n* (%)**	740				**<0.001**
Open		169 (22.8%)	50 (39.7%)	119 (19.4%)	
Laparoscopic		571 (77.2%)	76 (60.3%)	495 (80.6%)	
**Pathological tumor size, mm** ± SD	784	48.3 ± 29.3	68.1 ± 38.2	44.2 ± 25.3	**<0.001**
**pT stage, *n* (%)**	785				**<0.001**
1		495 (63.1%)	49 (36.3%)	446 (68.6%)	
2		52 (6.6%)	10 (7.4%)	42 (6.5%)	
3		231 (29.4%)	71 (52.6%)	160 (24.6%)	
4		7 (0.9%)	5 (3.7%)	2 (0.3%)	
**Surgical margins, *n* (%)**	785				0.09
Negative		753 (96%)	126 (93.4%)	627 (96.5%)	
Positive		32 (4%)	9 (6.6%)	23 (3.5%)	
**Fuhrman grade, *n* (%)**	775				**<0.001**
I		15 (1.9%)	0 (0%)	15 (2.4%)	
II		365 (47.1%)	32 (24.3%)	333 (52%)	
III		296 (38.2%)	65 (47.8%)	231 (36.1%)	
IV		99 (12.8%)	38 (27.9%)	61 (9.6%)	
**Histology, *n* (%)**	786				**<0.001**
Clear cell		583 (74.2%)	121 (89.6%)	462 (70.9%)	
Papillary		110 (14%)	10 (7.4%)	100 (15.4%)	
Chromophobe		63 (8%)	2 (1.5%)	61 (9.4%)	
Other		30 (3.8%)	2 (1.5%)	28 (4.3%)	
**Sarcomatoid features, *n* (%)**	772	75 (9.7%)	25 (19.4%)	50 (7.8%)	**<0.001**
**Micro vascular invasion, *n* (%)**	776	1113 (14.6%)	40 (30.5%)	73 (11.3%)	**<0.001**
**UISS score, n (%)**	774				**<0.001**
Low		249 (32.2%)	13 (9.6%)	236 (36.9%)	
Intermediate		430 (55.5%)	83 (61.5%)	347 (54.3%)	
High		95 (12.3%)	39 (28.9%)	56 (8.8%)	
**NLR at relapse or last follow-up** ± SD	485	3.4 ± 4.7	4.9 ± 3.7	2.9 ± 4.9	**<0.001**

**Table 2:** Sites of disease recurrence.

	*n*	Recurrence
**Recurrence localization, n (%)**	135	
Local		35 (25.9%)
Contralateral kidney		18 (13.3%)
Retroperitoneal lymph node		9 (6.7%)
Thoracic		70 (51.9%)
Bone		14 (10.4%)
Hepatic		8 (5.9%)
Cerebral		7 (5.2%)
Other		18 (13.3%)

**Table 3:** Uni- and multi-variate Cox proportional analysis of recurrence-free survival and baseline characteristics.

	Uni-Variate Analysis		Multi-Variate Analysis	
	HR (95% CI)	*p*-Value	HR (95% CI)	*p*-Value
**Gender**, male vs. female	1.03 [0.71–1.48]	0.8	0.80 [0.54–1.18]	0.3
**Age**, ≥61 vs. <61	1.51 [1.08–2.16]	**0.01**	1.32 [0.90–1.95]	0.1
**Pre-operative NLR**, ≥2.7 vs. <2.7	3.61 [2.56–5.13]	**<0.001**	2.89 [1.94–4.31]	**<0.001**
**Type of treatment**, radical vs. partial nephrectomy	2.42 [1.73–3.45]	**<0.001**	1.19 [0.76–1.87]	0.2
**Surgical approach**, laparoscopic vs. open	0.56 [0.40–0.81]	**0.002**	0.63 [0.43–0.92]	**0.02**
**pT stage**, 3–4 vs. 1–2	3.8 [2.69–5.35]	**<0.001**	1.80 [1.15–2.83]	**0.01**
**Fuhrman grade**, 3–4 vs. 1–2	3.15 [2.12–4.69]	**<0.001**	1.5 [0.92–2.43]	0.09
**Histology**, clear cell vs. other	3.12 [1.79–5.4]	**<0.001**	2.9 [1.1–7.8]	**0.02**
**Sarcomatoid features**	3.74 [2.40–5.83]	**<0.001**	1.89 [1.15–3.11]	**0.01**
**Micro-vascular invasion**	3.07 [2.12–4.47]	**<0.001**	1.10 [0.69–1.75]	0.6
**Surgical margins**, positive vs. negative	2.19 [1.11–4.32]	**0.02**	1.58 [0.75–3.3]	0.2

## Data Availability

Data presented are contained within the article; for additional information, datasets are also available upon request from the corresponding author.
